# Participation, perceived barriers, and course satisfaction in university physical education: a cross-sectional study of non-sport-major undergraduates at a Chinese university

**DOI:** 10.3389/fpsyg.2026.1931977

**Published:** 2026-09-09

**Authors:** Wanglong Yang, Boyi Yang, Xinding Zhang

**Affiliations:** School of Physical Education, Hainan Normal University, Haikou, Hainan, China

**Keywords:** China, course satisfaction, participation, perceived barriers, physical education, university students

## Abstract

University physical education (PE) is a structured learning context in which students’ participation and perceived barriers may be related to how they evaluate the course. This single-institution cross-sectional study examined associations among PE participation, perceived barriers, and PE course satisfaction among non-sport-major undergraduates at Hainan Normal University, China. Of 350 questionnaires distributed, 300 records retained after eligibility and data-quality screening were analyzed. Participants completed a researcher-developed 55-item Chinese questionnaire covering three participation domains, four satisfaction domains, and four barrier domains. The revised analysis evaluated the questionnaire using internal-consistency estimates, permutation-based parallel analysis, exploratory factor analysis, correlations, and HC3-robust regression with sensitivity analyses. Parallel analysis supported the intended three-, four-, and four-factor structures within this sample, but the instrument still requires independent psychometric confirmation. Higher reported PE participation was associated with higher course satisfaction, whereas greater perceived barriers were associated with lower satisfaction. After adjustment for the other variables included in the regression model, time constraints and lack of interest showed negative associations with satisfaction. These findings are sample-specific and do not establish causal or theory-specific mechanisms. Future multi-institutional studies using independently validated measures and longitudinal or intervention designs are needed.

## 1 Introduction

Regular physical activity supports physical and mental health, yet insufficient activity remains common during adolescence and young adulthood, including the transition to university (Bull et al., 2020). University students face academic demands, changing routines, and competing priorities that can make sustained activity difficult. Recent evidence syntheses identify time and other environmental constraints, social influences, and goal prioritization as important correlates of university students’ physical activity, while intervention reviews show potential benefits for wellbeing but also substantial variation in intervention effects and study quality (Pan et al., 2022; Brown et al., 2024; Huang et al., 2024; Liu et al., 2025).

University physical education (PE) provides a structured educational setting in which students can develop movement skills, experience physical activity, and form evaluations of teaching, facilities, assessment, and the course as a whole. Quality PE is expected to address physical, cognitive, social, and affective learning, and recent reviews indicate that PE-based approaches can support selected learning, fitness, and participation outcomes (Cale, 2021; Dudley et al., 2022; da Silva et al., 2022). Course satisfaction is therefore a relevant proximal indicator of students’ experience, although it should not be treated as a direct proxy for educational quality. Recent Chinese research has examined PE learning satisfaction and has also developed a multidimensional PE course-satisfaction measure, underscoring that students’ evaluations span multiple aspects of the course environment (Chen and Xiang, 2025; Jin and Xi, 2026).

Participation is related to student engagement, but the two terms are not interchangeable. Engagement frameworks distinguish behavioral, emotional, and cognitive components (Fredricks et al., 2004; Kahu and Nelson, 2018). In university PE, teacher support, autonomous motivation, and self-efficacy have been associated with student engagement, and motivationally supportive teaching has been linked with more favorable learning experiences (Guo et al., 2023; Leo et al., 2022; Guo et al., 2025). The participation instrument used in the present study includes attendance, effort, and consistency items as well as motivation, enjoyment, and perceived-benefit content. Accordingly, the present article uses the broader label PE participation and does not treat the 15-item composite as a pure or standardized measure of behavioral engagement.

Self-determination theory provides useful background for understanding why motivational experiences can matter in PE, particularly through autonomy, competence, relatedness, and the quality of motivational regulation (Deci and Ryan, 2000; Ryan and Deci, 2000). Systematic reviews support associations between need-supportive PE contexts, more autonomous motivation, and adaptive student outcomes (Vasconcellos et al., 2020; White et al., 2020). However, the present questionnaire did not measure basic psychological needs, autonomous or controlled regulation, or amotivation with theory-specific scales. Self-determination theory is therefore used only as contextual literature rather than as a model directly tested by this study.

Perceived barriers also operate across individual, interpersonal, and institutional levels. Reviews of university populations consistently identify limited time, academic pressure, resource constraints, social influences, and low motivation or interest as relevant barriers to physical activity (Pan et al., 2022; Silva et al., 2022; Brown et al., 2024). Recent Chinese evidence similarly emphasizes multilevel influences on campus fitness engagement, while university surveys continue to identify time and motivational barriers as prominent concerns (Elmahgoub et al., 2025; Fang and Liu, 2026). Peer-related concerns may also affect participation through perceived judgment, belonging, and body-related discomfort (Van den Berghe et al., 2021; Haug et al., 2023). Examining separate barrier domains can therefore be more informative than relying only on a global barrier score.

The specific empirical gap is narrower than a general lack of research on Chinese university PE. Recent Chinese studies have examined physical literacy and motivation in relation to broader wellbeing (Wang et al., 2023), teacher support in relation to engagement (Guo et al., 2025), and teacher care, enjoyment, and self-efficacy in relation to PE learning satisfaction (Chen and Xiang, 2025). In addition, a recently developed Chinese PE course-satisfaction scale has provided substantially stronger psychometric evidence for measuring satisfaction than was available when the present questionnaire was created (Jin and Xi, 2026). What remains less clear is whether students’ broad course-specific participation and distinct perceived barriers are simultaneously associated with their evaluation of compulsory university PE, particularly among non-sport-major undergraduates. Existing barrier studies also more often address general physical activity than satisfaction with the PE course itself.

Accordingly, this study examined PE participation, perceived barriers, and course satisfaction among non-sport-major undergraduates at Hainan Normal University, China. The study addressed three questions: (1) What are the descriptive levels, subscale internal consistency, and preliminary within-sample factor structure of the participation, satisfaction, and barrier measures? (2) How are the broad participation, perceived-barrier, and course-satisfaction composites associated? (3) Which barrier domains remain associated with course satisfaction after statistical adjustment for participation and the other barrier domains included in the model? The study was designed to estimate cross-sectional associations rather than causal effects.

## 2 Materials and methods

### 2.1 Study design and reporting

This was a single-institution cross-sectional observational survey with the individual student as the unit of analysis. The manuscript was prepared in line with STROBE guidance for cross-sectional research. The data were originally collected for the first author’s doctoral dissertation (Yang, 2025). The dissertation addressed broader descriptive, comparative, and curriculum-development questions and reported descriptive medians, Friedman comparisons among domains, and bivariate Spearman associations. The present article asks a narrower question focused on course satisfaction and adds a psychometric reanalysis (parallel analysis and exploratory factor analysis), composite-score evaluation, HC3-robust multivariable regression, model diagnostics, and sensitivity analyses. No dissertation table is reproduced verbatim: Tables 1–4 in this article were newly constructed from the archived item-level data for the present analyses, and the regression and sensitivity findings were not reported as the main analyses in the dissertation. Because the institution itself was not the unit of analysis and no case-study methodology was used, the design is described as a single-institution cross-sectional survey rather than an institutional case study.

**Table 1:** Descriptive statistics and internal consistency for the study measures (*n* = 300).

Measure	Items	Cronbach’s *α*	*M*	*M* (SD)	McDonald’s *ω*
PE participation	15	–	1.87	0.61	–
Participation intensity	5	0.888	1.66	0.66	0.894
Motivation for participation	5	0.953	2.08	0.98	0.954
Participation consistency	5	0.861	1.86	0.69	0.869
PE course satisfaction	20	–	2.09	0.66	–
Teaching equipment	5	0.953	2.14	0.89	0.954
Teaching venue	5	0.942	2.29	0.86	0.943
Teaching methods	5	0.965	1.91	0.78	0.965
Evaluation system	5	0.972	2.02	0.87	0.972
Perceived barriers	20	–	3.67	0.63	–
Physical limitations	5	0.871	3.54	0.85	0.875
Time constraints	5	0.885	3.65	0.79	0.889
Lack of interest	5	0.912	3.98	0.75	0.913
Peer-related barriers	5	0.885	3.48	0.83	0.886

Scores are mean item scores on five-point response scales. Internal-consistency coefficients are reported for the intended subscales; coefficients for the multidimensional total composites are omitted because they are not interpreted as evidence of unidimensionality.

**Table 2:** Parallel analysis and preliminary exploratory factor-analytic evidence for the three item sets (*n* = 300).

Scale	Factors (intended/PA)	KMO	Bartlett’s *χ^2^* (df)	Primary loading range	Factor-correlation range	Mean communality
Participation	3/3	0.881	3683.65 (105)	0.520–0.952	0.363–0.451	0.686
Satisfaction	4/4	0.929	7471.76 (190)	0.794–0.970	0.350–0.629	0.826
Perceived barriers	4/4	0.913	3935.96 (190)	0.627–0.873	0.420–0.535	0.632

PA, parallel analysis. Principal-axis factoring with oblimin rotation. Parallel analysis used 1,000 independent column permutations and the 95th-percentile random-eigenvalue criterion. All Bartlett’s tests *p* < 0.001. Mean communality is the mean extracted item communality, not the percentage of total variance explained. Complete rotated pattern matrices and factor-correlation matrices are provided in Supplementary Tables S5–S7.

**Table 3:** Spearman correlations among the broad participation, course-satisfaction, and perceived-barrier composites (*n* = 300).

Variable	*M* (SD)	1	2	3
1. PE participation	1.87 (0.61)	–		
2. PE course satisfaction	2.09 (0.66)	0.609	–	
3. Perceived barriers	3.67 (0.63)	−0.503	−0.480	–

Entries are Spearman’s *ρ*. All *p* < 0.001 (two-sided).

**Table 4:** HC3-robust ordinary least squares models of PE course satisfaction (*n* = 300).

Predictor	Model 1 *b*	95% CI	*β*	*p*	Model 2 *b*	95% CI	*β*	*p*
Intercept	2.001	[1.460, 2.541]	–	<0.001	2.350	[1.741, 2.959]	–	<0.001
PE participation	0.524	[0.413, 0.635]	0.485	<0.001	0.472	[0.351, 0.592]	0.437	<0.001
Overall perceived barriers	−0.243	[−0.352, −0.134]	−0.232	<0.001	–	–	–	–
Physical limitations	–	–	–	–	0.077	[−0.039, 0.194]	0.099	0.194
Time constraints	–	–	–	–	−0.180	[−0.288, −0.073]	−0.215	0.001
Lack of interest	–	–	–	–	−0.182	[−0.310, −0.055]	−0.207	0.005
Peer-related barriers	–	–	–	–	−0.009	[−0.105, 0.088]	−0.011	0.861
*R^2^*	0.402				0.440			
Adjusted *R^2^*	0.398				0.430			

Outcome = broad PE course-satisfaction composite. HC3 robust standard errors were used. *β* = standardized coefficient. Model 1 robust *F* (2, 297) = 107.90, *p* < 0.001. Model 2 robust *F* (5, 294) = 52.11, *p* < 0.001. Predictor VIFs in Model 2 ranged from 1.44 to 1.94. The estimates are adjusted only for variables included in the model and not for unavailable demographic or background covariates.

### 2.2 Setting, participants, and procedures

The study was conducted at Hainan Normal University, Hainan Province, China. The target population was first- and second-year undergraduates enrolled in non-sport majors. The original study documentation describes a non-probability quota approach by year level. Accessible first- and second-year PE classes were used for class-based convenience recruitment rather than probability selection of classes or individual students. Students were approached in the teaching context and invited to complete an anonymous questionnaire through an online survey link shared via WeChat and through in-person administration. Data collection took place from January 10 to July 1, 2025, after ethical approval had been obtained. The retained documentation does not preserve the number of questionnaires completed through each administration channel or the original survey-platform settings used to prevent duplicate online submissions. A total of 350 questionnaires were distributed. During revision, the research team recovered and verified a record-level exclusion log covering all 50 nonretained questionnaires. Each record was assigned one primary exclusion reason: incomplete questionnaire or missing key questions (*n* = 12), abnormally short completion time documented during the original screening process (*n* = 10), pronounced straight-lining or extremely low response variability (*n* = 8), duplicate submission (*n* = 6), failure to meet the eligibility criteria (*n* = 6), clearly mechanical or patterned responding (*n* = 4), irreconcilable screening inconsistencies or otherwise invalid records (*n* = 2), and other incomplete or unusable records, including withdrawal/incomplete consent or record anomalies (*n* = 2). The remaining 300 records constituted the analytical sample (retention proportion = 85.7%). The de-identified analytical file contains only the retained substantive item responses and does not contain the excluded records or the original screening paradata; therefore, the present reanalysis reports the verified historical exclusion log but does not retrospectively re-estimate or alter the original screening thresholds. A de-identified summary of the exclusion log is provided in Supplementary Table S12.

The de-identified item-level archive used for the present reanalysis does not retain person-level demographic fields and therefore cannot link demographic characteristics to questionnaire responses or use them as regression covariates. Aggregate dissertation documentation describes the retained sample as 150 men and 150 women and as 150 first-year and 150 second-year students. The available records do not document why the demographic fields were omitted when the de-identified analytical archive was created. Because those fields are not present in the item-level file, the aggregate counts cannot be independently cross-checked against individual responses, and no analyses stratified or adjusted by demographic characteristics were conducted.

### 2.3 Questionnaire development and Chinese administration

The instrument was researcher-developed, drafted initially in English for the doctoral study, and administered in Chinese. Before formal administration, three education experts at Cavite State University reviewed the instrument for clarity, relevance, and appropriateness, and a pilot test was conducted with 30 students who were similar to, but not part of, the final sample. The available documentation does not preserve translator identities, an independent forward-backward translation record, or item-level content-validity indices; therefore, the instrument is treated as a context-specific measure rather than a formally cross-culturally validated questionnaire. The original protocol defined the semantic scoring key as 5 = strongly agree and 1 = strongly disagree for participation and barriers, and 5 = highly satisfied and 1 = highly dissatisfied for satisfaction. A revision-stage scoring audit compared the English protocol, archived Chinese questionnaire screenshots, and the 55-item analytical tabulation. The screenshots confirmed that response labels were displayed in different left-to-right orders across blocks; some blocks ran from positive to negative and others from negative to positive. The analytical scoring was defined by the meaning of the selected response category rather than by its left-to-right screen position, and no *post hoc* reversal was applied solely because a block was displayed in the opposite visual order. Supplementary Table S3 documents the observed display order and intended semantic coding for each block. Because the original survey-platform code map is no longer archived, this audit cannot independently reconstruct the platform’s historical button-to-code mapping; that residual uncertainty is acknowledged explicitly as a methodological limitation.

### 2.4 Measures

PE participation was assessed with 15 items across three five-item domains: participation intensity, motivation for participation, and consistency of participation. The content included attendance, active effort, regular involvement, enjoyment, perceived benefits, and reasons for participation. Domain scores and a broad participation composite were calculated as item means. Because the motivation domain contains affective and evaluative content, the total score is referred to as PE participation rather than behavioral engagement. It is not interpreted as a unidimensional latent engagement construct.

PE course satisfaction was assessed with 20 items across four five-item domains: teaching equipment, teaching venue, teaching methods, and the evaluation system. Items evaluated course resources, facilities, pedagogical practices, and assessment arrangements. Domain scores and a broad course-satisfaction composite were calculated as item means.

Perceived barriers were assessed with 20 items across four five-item domains: physical limitations, time constraints, lack of interest, and peer-related factors. Higher scores indicated stronger perceived obstacles to PE participation. Domain scores and a broad perceived-barrier composite were calculated as item means. The three broad composites were retained as simple equal-weight summaries of related course facets for descriptive and association analyses; they were not treated as unidimensional latent scales. Evidence relevant to this pragmatic aggregation was examined through factor correlations, Spearman correlations among the intended domains, a sensitivity model replacing the participation composite with its three domain scores, and additional models repeating Model 2 separately for the four satisfaction domains. The substantive barrier-specific interpretation relies primarily on Model 2 rather than on the global barrier score. Item-level wording and scoring are provided in the Supplementary material.

### 2.5 Statistical analysis

All analyses were conducted in Python 3.11 using NumPy, SciPy, and statsmodels. The participant was the unit of analysis. Means, standard deviations, observed ranges, and subscale internal-consistency estimates were calculated. Cronbach’s *α* and McDonald’s *ω* were reported for the intended subscales; coefficients for the multidimensional total composites were not used as evidence that the total scores represented single latent dimensions (Cronbach, 1951; McDonald, 1999).

Separate exploratory factor analyses were conducted for the participation, satisfaction, and barrier item sets using principal-axis factoring with oblimin rotation. To avoid relying only on the prespecified number of factors, factor retention was also examined with permutation-based parallel analysis using 1,000 iterations; observed correlation-matrix eigenvalues were compared with the 95th-percentile eigenvalues from independently permuted item columns. The prespecified three-, four-, and four-factor solutions were then examined because the parallel analysis supported those factor counts. KMO statistics, Bartlett’s tests of sphericity, complete rotated pattern matrices, cross-loadings, factor correlations, and mean extracted communalities were evaluated. Complete item-level pattern matrices and factor-correlation matrices are reported in Supplementary Tables S5–S7. Mean communality is reported as the average item communality and is not described as the percentage of total variance explained. These analyses are within-sample exploratory evidence and do not constitute independent confirmatory validation, cross-cultural validation, test–retest reliability, or measurement-invariance testing.

Spearman rank-order correlations were used for the primary bivariate analyses because the broad composite scores were derived from ordinal responses and their empirical distributions were not assumed to be normal. Distributional characteristics were examined using skewness, kurtosis, Shapiro–Wilk tests, and graphical inspection. All correlation coefficients are reported as Spearman’s *ρ*.

Two ordinary least squares (OLS) models were estimated with the 20-item course-satisfaction composite as the outcome. Model 1 included the broad participation and perceived-barrier composites. Model 2 retained participation and replaced the global barrier composite with physical limitations, time constraints, lack of interest, and peer-related barriers. OLS was used because the 20-item mean score had 52 observed values and was treated as an approximately continuous composite; inference used HC3 heteroscedasticity-consistent standard errors. Model diagnostics included residual distribution and Q-Q inspection, residual-versus-fitted patterns, Breusch-Pagan and White heteroscedasticity tests, variance inflation factors, and Cook’s distance. Sensitivity analyses included 3,000 nonparametric bootstrap resamples for coefficient confidence intervals, Huber *M*-estimation, median quantile regression, a domain-level model replacing the participation composite with participation intensity, motivation, and consistency, and four additional HC3 models using each satisfaction domain as the outcome. Spearman correlations among the intended subdomains were also calculated to describe their relatedness. An exploratory unrotated single-factor diagnostic across all 55 items was calculated only to describe whether one dominant factor accounted for most item variance; it was not treated as a valid correction for common-method bias. Because no demographic or background covariates were available in the item-level archive, the regression coefficients are associations adjusted only for the variables included in each model and should not be interpreted as causal or fully confounder-adjusted effects.

### 2.6 Ethics statement

Ethical approval for the doctoral research protocol was obtained from the Cavite State University Ethics Review Board (Study No. CvSU-ERB-SSR-2024-0106 V1; approved January 7, 2025) before data collection. As the degree-granting institution for the doctoral research, Cavite State University served as the reviewing ethics body for the doctoral protocol. Hainan Normal University granted institutional permission for the researcher to approach students and administer the questionnaire in the university teaching context; the university did not issue a separate local ethics-committee approval for this study. The manuscript therefore distinguishes site authorization from ethical review. Participants were informed of the study purpose, the voluntary nature of participation, confidentiality procedures, their right to stop before submission, and that participation or non-participation would not affect academic performance. Informed consent was obtained from all participants.

## 3 Results

### 3.1 Sample retention and data completeness

Of the 350 questionnaires distributed during the study period, 50 were excluded according to the verified record-level screening log and 300 were retained for analysis (retention proportion = 85.7%). The primary exclusion reasons were incomplete questionnaire or missing key questions (*n* = 12), abnormally short completion time (*n* = 10), pronounced straight-lining or extremely low response variability (*n* = 8), duplicate submission (*n* = 6), failure to meet eligibility criteria (*n* = 6), clearly mechanical or patterned responding (*n* = 4), irreconcilable screening inconsistencies or invalid records (*n* = 2), and other incomplete or unusable records (*n* = 2) (Supplementary Table S12). There were no missing responses across the 55 substantive items in the 300 retained records. Aggregate dissertation documentation reports 150 men and 150 women and an equal split of 150 first-year and 150 second-year students, but these demographic fields were not retained in the de-identified item-level archive and therefore could not be linked to outcomes or included as covariates.

### 3.2 Descriptive statistics and internal consistency

Table 1 summarizes the descriptive statistics and intended-subscale internal consistency. Across the 11 intended subscales, Cronbach’s *α* ranged from 0.861 to 0.972 and McDonald’s *ω* ranged from 0.869 to 0.972. The multidimensional total composites are therefore presented descriptively rather than using a single reliability coefficient as evidence for unidimensionality. Mean PE participation was 1.87 (SD = 0.61), mean course satisfaction was 2.09 (SD = 0.66), and mean perceived barriers were 3.67 (SD = 0.63) on the five-point scales. Among the participation domains, motivation had the highest mean (*M* = 2.08, SD = 0.98); among satisfaction domains, teaching venue had the highest mean (*M* = 2.29, SD = 0.86); and among barrier domains, lack of interest had the highest mean (*M* = 3.98, SD = 0.75). The revision-stage scoring audit reproduced these composite values from the archived 55-item tabulation under the protocol-defined semantic coding and did not identify any item requiring reversal solely because of visual response-order direction.

### 3.3 Preliminary structural evidence

Table 2 summarizes the within-sample structural evidence. Permutation-based parallel analysis supported three participation factors, four satisfaction factors, and four barrier factors, matching the intended dimensional structure. KMO values were 0.881, 0.929, and 0.913, respectively, and Bartlett’s tests were significant for all three item sets (all *p* < 0.001). In the corresponding principal-axis solutions with oblimin rotation, primary pattern loadings ranged from 0.520 to 0.952 for participation, 0.794 to 0.970 for satisfaction, and 0.627 to 0.873 for barriers; no secondary loading exceeded 0.30. Factor correlations were 0.363–0.451 for participation, 0.350–0.629 for satisfaction, and 0.420–0.535 for barriers, indicating related but distinguishable domains. Spearman correlations among the intended domain scores were likewise positive and moderate: 0.583–0.590 for participation, 0.495–0.677 for satisfaction, and 0.440–0.540 for barriers (Supplementary Table S10). Mean extracted communalities were 0.686, 0.826, and 0.632, respectively. Complete pattern matrices and factor correlations are provided in Supplementary Tables S5–S7. These findings support related multidimensional domains within this sample but do not independently validate the questionnaire or establish that the broad composites are unidimensional latent scales.

### 3.4 Bivariate associations

As shown in Table 3, PE participation was positively associated with course satisfaction (*ρ* = 0.609, *p* < 0.001). Perceived barriers were negatively associated with participation (*ρ* = −0.503, *p* < 0.001) and with course satisfaction (*ρ* = −0.480, *p* < 0.001).

### 3.5 Regression models of course satisfaction

Table 4 presents the HC3-robust OLS models. In Model 1, the broad participation and perceived-barrier composites accounted for 40.2% of the observed variance in course satisfaction (*R^2^* = 0.402; adjusted *R^2^* = 0.398). Participation was positively associated with satisfaction (*b* = 0.524, 95% *CI* [0.413, 0.635], *β* = 0.485, *p* < 0.001), whereas the broad barrier composite was negatively associated with satisfaction (*b* = −0.243, 95% *CI* [−0.352, −0.134], *β* = −0.232, *p* < 0.001). These are model-adjusted associations and are not adjusted for demographic or background confounders.

**Figure 1:**
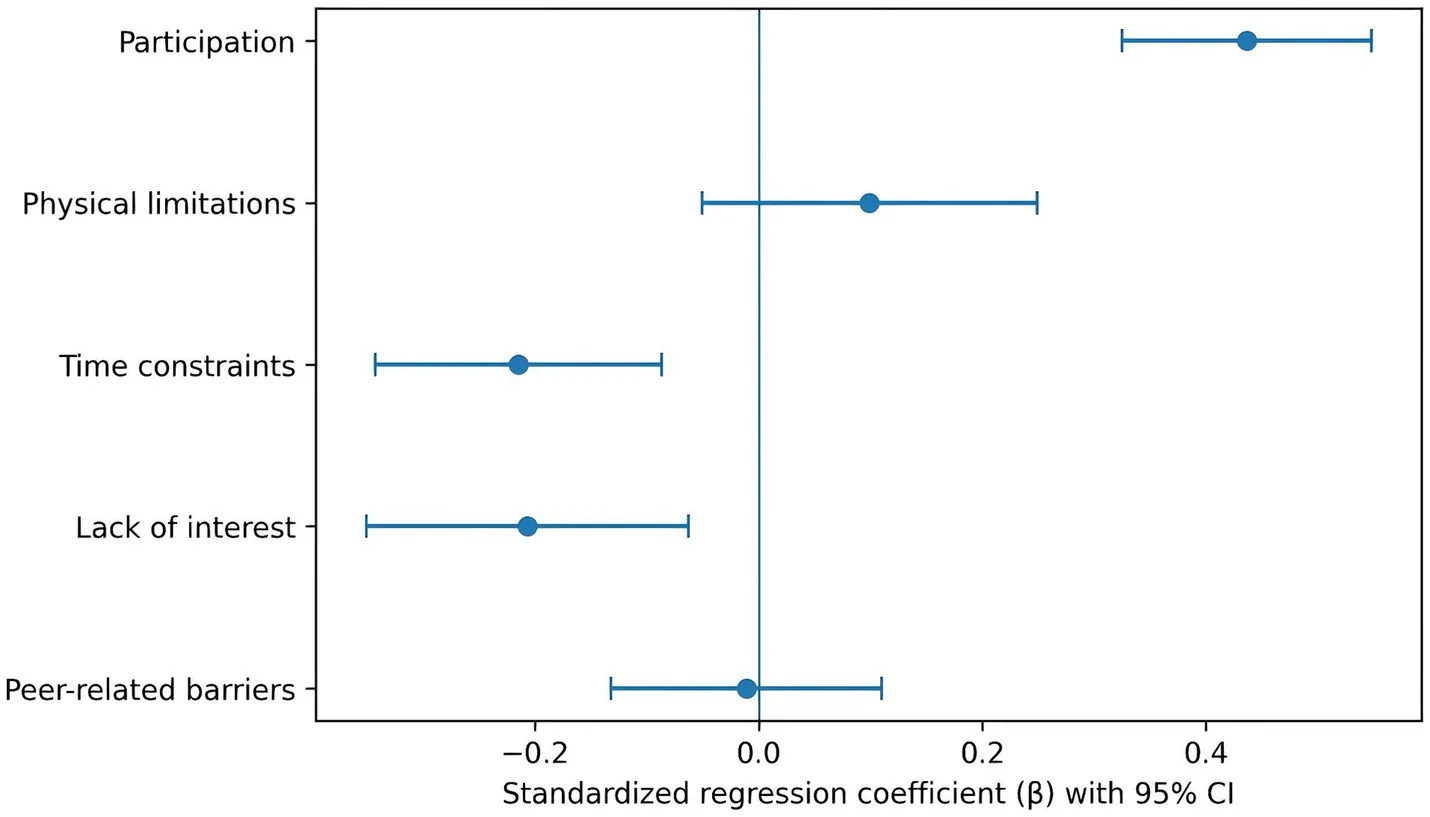
Standardized regression coefficients (*β*) and 95% confidence intervals from Model 2 with PE course satisfaction as the outcome. The model included the broad PE participation composite and the four perceived-barrier domains (*n* = 300). HC3 robust standard errors were used. Coefficients represent associations adjusted only for the other variables shown in the model; they are not causal effects.

In Model 2, participation and the four barrier domains accounted for 44.0% of the observed variance in course satisfaction (*R^2^* = 0.440; adjusted *R^2^* = 0.430). Participation remained positively associated with satisfaction after adjustment for the four included barrier domains (*b* = 0.472, 95% *CI* [0.351, 0.592], *β* = 0.437, *p* < 0.001). Time constraints (*b* = −0.180, 95% *CI* [−0.288, −0.073], *β* = −0.215, *p* = 0.001) and lack of interest (*b* = −0.182, 95% *CI* [−0.310, −0.055], *β* = −0.207, *p* = 0.005) were negatively associated with satisfaction after adjustment for the other variables included in the model. Physical limitations (*b* = 0.077, 95% *CI* [−0.039, 0.194], *p* = 0.194) and peer-related barriers (*b* = −0.009, 95% *CI* [−0.105, 0.088], *p* = 0.861) were not statistically distinguishable from zero in this model. Figure 1 displays the standardized coefficients.

### 3.6 Model diagnostics and sensitivity analyses

All three broad composites departed from strict normality by Shapiro–Wilk testing (participation *p* < 0.001; satisfaction *p* < 0.001; barriers *p* = 0.046), although distributional departures were modest (skewness = 0.262, 0.214, and −0.107; excess kurtosis = −0.445, 0.284, and −0.098, respectively). For Model 2, residuals showed positive skew (0.593) and excess kurtosis (1.529), and the Shapiro–Wilk test was significant (*p* < 0.001). The Breusch-Pagan test did not indicate heteroscedasticity (*p* = 0.177), whereas the White test did (*p* = 0.001), supporting retention of HC3 robust standard errors. Predictor VIFs ranged from 1.44 to 1.94. The maximum Cook’s distance was 0.175; 15 observations exceeded the 4/*N* heuristic, but no observation approached a Cook’s distance of 1. Sensitivity analyses were directionally consistent with the primary model. The 3,000-resample bootstrap 95% CIs excluded zero for participation [0.353, 0.589], time constraints [−0.289, −0.081], and lack of interest [−0.298, −0.046], while including zero for physical limitations and peer-related barriers. Huber *M*-estimation and median regression produced the same coefficient directions and the same significance pattern for participation, time constraints, and lack of interest. When the participation composite was replaced by its three domains, participation intensity, motivation, and consistency were each positively associated with satisfaction, while time constraints and lack of interest remained negatively associated with satisfaction (Supplementary Table S9). In models using the four satisfaction domains separately, participation was positively associated with all four outcomes; time constraints were negatively associated with venue and teaching-method satisfaction, whereas the estimates for lack of interest were negative in all four models but their individual 95% CIs included zero (Supplementary Table S11). Thus, the broad-score results should not be interpreted as implying identical barrier patterns across every satisfaction facet. As an exploratory common-method diagnostic, the first unrotated factor across all 55 items accounted for 33.0% of total item variance; this does not rule out shared-method inflation and is not interpreted as evidence that common-method bias is absent.

## 4 Discussion

### 4.1 Principal findings

This single-institution cross-sectional study examined broad PE participation, perceived barriers, and course satisfaction using a researcher-developed questionnaire. Three findings summarize the observed associations. First, students reporting greater PE participation also tended to report greater course satisfaction. Second, greater perceived barriers were associated with lower satisfaction. Third, after adjustment for participation and the other barrier domains included in Model 2, time constraints and lack of interest showed negative associations with satisfaction, whereas physical limitations and peer-related barriers did not. These findings should be interpreted as sample-specific associations rather than as causal effects or evidence that any barrier is an established intervention target.

### 4.2 Participation and course satisfaction

The positive association between PE participation and course satisfaction is compatible with prior evidence linking supportive PE environments, motivation, and engagement-related experiences (Guo et al., 2023; Leo et al., 2022; Guo et al., 2025). However, interpretation requires care because the participation composite is broader than behavioral engagement. Its intensity and consistency items primarily describe attendance, effort, and regular involvement, whereas the motivation domain includes enjoyment, perceived wellbeing benefits, and reasons for participating. Satisfaction items, in contrast, evaluate equipment, venue, teaching methods, and assessment. Thus, the item content is not identical across constructs, but the motivational and evaluative content within the participation measure may create some conceptual overlap with students’ broader evaluations of the course and could contribute to the magnitude of the observed association.

Self-determination theory offers a possible background framework for interpreting motivational experiences in PE, but the present study does not test its mechanisms. The questionnaire did not assess autonomy, competence, relatedness, autonomous regulation, controlled regulation, or amotivation with established theory-specific scales. Accordingly, the observed participation-satisfaction association should not be attributed to need satisfaction or a specific form of motivational regulation. Recent Chinese research that directly measured teacher care, exercise enjoyment, self-efficacy, and PE learning satisfaction demonstrates the type of theory-linked measurement that would be required to test those mechanisms more directly (Chen and Xiang, 2025).

Reverse directionality and uncontrolled confounding are also plausible. Students who are more satisfied may participate more, while prior sport experience, physical competence, instructor characteristics, course type, health status, and demographic or scheduling factors may influence both constructs. Because these variables were not available in the de-identified archive, the regression estimates cannot isolate an explanatory effect of participation. The results therefore support covariation between broad participation and course satisfaction within this sample, not a directional pathway.

### 4.3 Why time constraints and lack of interest mattered

Time constraints were negatively associated with course satisfaction after adjustment for the other variables included in Model 2. This pattern is consistent with systematic reviews identifying time and competing academic demands as common barriers to university students’ physical activity (Pan et al., 2022; Brown et al., 2024). However, the present questionnaire measured the perceived strength of time constraints rather than their specific sources. It therefore cannot determine whether students were responding to academic workload, timetable conflicts, commuting, employment, extracurricular commitments, or other demands. Those possibilities come from prior literature and should not be treated as qualitative findings from the present quantitative survey. Longitudinal or intervention research would be required to determine whether reducing perceived time constraints changes PE course satisfaction.

Lack of interest was also negatively associated with satisfaction after adjustment for the other variables included in the model. Recent work continues to identify low motivation or interest as an important barrier to university students’ activity (Elmahgoub et al., 2025; Fang and Liu, 2026). In the present study, however, lack of interest is a broad self-reported barrier rather than a measure of intrinsic motivation, autonomous regulation, controlled regulation, or amotivation. The association is therefore best interpreted at the level directly measured: students who endorsed stronger lack-of-interest barriers also tended to report lower course satisfaction. The data do not identify which motivational mechanism, if any, produced that association.

The coefficients for physical limitations and peer-related barriers were not statistically distinguishable from zero after adjustment for the other variables included in Model 2. This does not imply that those barriers are unimportant. They may be relevant to specific students or operate through outcomes not captured by the broad satisfaction composite. Prior research links body-related concerns, social comparison, and supportive or thwarting interpersonal climates with PE participation and engagement (Van den Berghe et al., 2021; Haug et al., 2023). Future studies should use validated domain-specific measures and examine whether associations vary by sex or gender, health status, prior sport experience, course type, and other characteristics unavailable here.

### 4.4 Implications for university PE practice

The present findings generate hypotheses for future intervention research rather than demonstrate that specific practice changes will improve satisfaction. One testable hypothesis is that PE designs offering appropriate task differentiation, meaningful activity choice, clear progress feedback, and supportive instructional structure may improve students’ course experiences. Such approaches are consistent with evidence on autonomy-supportive and structured teaching, but they should be prospectively evaluated rather than inferred to be effective from the present cross-sectional associations (Guo et al., 2023; Patzak and Zhang, 2025).

A second hypothesis concerns time-related friction. Future trials or natural experiments could test whether predictable scheduling, transparent make-up arrangements, modular course organization, or technology-supported preparation reduce perceived time barriers without weakening learning outcomes. University-level reviews suggest that technology-supported and flexible approaches can be feasible strategies for promoting activity, but their effect on compulsory PE course satisfaction remains an empirical question (Lynch et al., 2022; Johannes et al., 2024).

A third hypothesis is that curriculum relevance and activity variety may matter for students reporting low interest. Future studies could test whether diversified content, informed choice, health-literacy content, transferable self-management skills, or progress-oriented assessment improve both participation and course satisfaction. These approaches are compatible with health-based PE principles, but the present data do not establish that implementing them would change the observed outcomes (Fernandez-Rio and Quennerstedt, 2022; Zhang et al., 2024).

### 4.5 Strengths and limitations

The study has several strengths. It analyzes a complete 55-item dataset for 300 retained participants, reports multidomain participation, satisfaction, and barrier measures, and distinguishes total barriers from specific barrier domains. The revised analysis supplements the prespecified exploratory factor solutions with permutation-based parallel analysis and complete item-level pattern matrices. HC3 robust standard errors, bootstrap confidence intervals, Huber estimation, median regression, and a domain-level participation sensitivity model provide multiple checks on the primary regression pattern. The study also focuses on non-sport-major undergraduates in a Chinese compulsory-PE context, while framing its contribution as context-specific rather than nationally representative.

The limitations are substantial. First, the cross-sectional design prevents causal inference and cannot establish whether participation precedes satisfaction or vice versa. Second, the sample came from one university and used non-probability, class-based quota/convenience recruitment, limiting external generalizability. Third, although the dissertation documentation provides aggregate sex and year-level counts, the de-identified item-level archive contains no demographic or background variables. The models therefore could not adjust for age, sex or gender, discipline, health status, socioeconomic circumstances, prior sport experience, instructor, or course type, and the reported coefficients remain vulnerable to uncontrolled confounding (Heredia-León et al., 2023). Fourth, although the recovered and verified exclusion log now documents the primary reason for each of the 50 nonretained questionnaires, the excluded records and the original screening paradata are not part of the de-identified analytical archive. The present reanalysis therefore cannot independently rerun the historical screening decisions or evaluate the effect of alternative screening thresholds. This is a residual reproducibility limitation, although the sample flow and exclusion categories are now explicitly documented. Because recruitment occurred in PE teaching contexts at the authors’ institution, perceived authority or social-desirability effects also cannot be completely excluded despite the study’s voluntariness and confidentiality procedures.

Fifth, all substantive variables were self-reported in the same questionnaire at the same time. Recall, acquiescence, evaluation tendency, and other shared-method processes could inflate or distort associations, particularly because the participation composite includes motivational and evaluative content. The dataset contains no temporal separation, external criterion, marker variable, or multi-informant measurement that would permit a strong statistical correction for common-method variance. The exploratory single-factor diagnostic does not eliminate this concern; future work should combine validated self-report measures with objective attendance, observational indicators, device-based activity, instructor or course characteristics, or temporally separated measurement. Sixth, the questionnaire was researcher-developed and translated from an English draft without a preserved independent forward-backward translation record, translator credentials, item-level content-validity indices, test–retest reliability, independent CFA, or measurement-invariance testing. The Chinese form also used different left-to-right response-option orders across blocks. The revision-stage audit confirmed the intended semantic scoring used in the archived tabulation, but the original platform-level button-to-code mapping is no longer available for independent reconstruction. The unusually low participation and satisfaction means and high barrier means must therefore be interpreted with this residual scoring uncertainty in mind. Finally, the instrument does not directly measure self-regulated learning or established self-determination-theory constructs. The findings should be interpreted as context-specific evidence about broad PE participation, perceived barriers, and course satisfaction rather than as a direct test of those theories.

The dataset originated in the first author’s doctoral research. The relationship between the dissertation and this article is described in the Methods and Acknowledgments: the dissertation emphasized descriptive summaries, Friedman comparisons, bivariate Spearman associations, and curriculum enhancement, whereas the present article focuses on course-satisfaction associations and adds parallel analysis, exploratory factor reporting, multivariable HC3 regression, model diagnostics, and sensitivity analyses. No dissertation table is reproduced verbatim in this manuscript (Yang, 2025).

## 5 Conclusion

Among 300 non-sport-major undergraduates at one Chinese university, higher broad PE participation was associated with higher course satisfaction, while greater perceived barriers were associated with lower satisfaction. After adjustment for the other variables included in the regression model, time constraints and lack of interest showed negative associations with satisfaction in this sample. These results are contingent on a single-institution, cross-sectional, self-report dataset and a researcher-developed questionnaire whose factor structure and scoring require independent confirmation. The participation composite also contains behavioral and motivational content, and the study did not measure the psychological needs, motivational regulations, or self-regulatory processes required for a direct test of self-determination or self-regulated-learning theory. Accordingly, the findings should be treated as hypothesis-generating evidence for future multi-institutional, longitudinal, and intervention research rather than as evidence that changing participation or specific barriers will necessarily improve PE course satisfaction.

## Data Availability

The original contributions presented in the study are included in the article/Supplementary material, further inquiries can be directed to the corresponding authors.
