# Supplementary material for: Participation, perceived barriers, and course satisfaction in university physical education: a cross-sectional study of non-sport-major undergraduates at a Chinese university
Source: Front Psychol. 2026 Sep 9;17:1931977. doi: 10.3389/fpsyg.2026.1931977 (PMC13598082; doi:10.3389/fpsyg.2026.1931977)
Supplement: Supplementary file 1 [file Supplementary_file_1.DOCX]

**Supplementary Material
Bilingual Item Codebook and Fielded Chinese Questionnaire Items**

**Supplementary Table S1.** Item-level Chinese wording, English reporting translation, construct assignment, and scoring key.

Scoring key. For participation and perceived-barrier items, the original protocol used 5 = Strongly agree, 4 = Agree, 3 = Neutral, 2 = Disagree, and 1 = Strongly disagree. For satisfaction items, the original protocol used 5 = Highly satisfied, 4 = Satisfied, 3 = Neutral, 2 = Dissatisfied, and 1 = Highly dissatisfied. Higher scores indicated more of the named construct. The Chinese online form displayed response labels in different left-to-right orders across blocks; the numerical coding described here follows the original English protocol and the archived analytic tabulation file.

| **Domain** | **Item** | **Chinese fielded wording** | **English reporting translation** | **Scoring key** |
| --- | --- | --- | --- | --- |
| Participation intensity | PI1 | 作为我的课程的一部分，我定期参加我的体育课。 | I regularly attend my physical education classes as part of my program. | 5 = Strongly agree; 1 = Strongly disagree |
| Participation intensity | PI2 | 在体育课上，我积极地参加运动和练习，不管它们是否与我的专业一致。 | During physical education classes, I actively participate in activities and exercises, regardless of whether they align with my major. | 5 = Strongly agree; 1 = Strongly disagree |
| Participation intensity | PI3 | 我在体育课程中不断地努力，尽力成为重要的团队成员。 | I put sustained effort into physical education sessions and try to be a valuable member of the group. | 5 = Strongly agree; 1 = Strongly disagree |
| Participation intensity | PI4 | 我充分从事体育活动，了解它们的重要性，尽管我的专业不同。 | I engage fully in physical education activities and recognize their importance despite my major. | 5 = Strongly agree; 1 = Strongly disagree |
| Participation intensity | PI5 | 我致力于通过积极参与体育教育来改善我的身体素质。 | I am committed to improving my physical fitness through active participation in physical education. | 5 = Strongly agree; 1 = Strongly disagree |
| Motivation for participation | PM1 | 我有动力参加体育课程，因为我相信它有助于我的整体健康。 | I am motivated to participate in physical education classes because I believe they contribute to my overall well-being. | 5 = Strongly agree; 1 = Strongly disagree |
| Motivation for participation | PM2 | 我在体育课中获得的技能和知识激励着我积极参与其中。 | The skills and knowledge I gain in physical education classes motivate me to participate actively. | 5 = Strongly agree; 1 = Strongly disagree |
| Motivation for participation | PM3 | 我参加体育课程是因为我喜欢体育活动，即便它们与我的专业无关。 | I participate in physical education classes because I enjoy the physical activities, even when they are unrelated to my major. | 5 = Strongly agree; 1 = Strongly disagree |
| Motivation for participation | PM4 | 体育课上的社会互动和团队合作激励着我积极地参与进来。 | The social interaction and teamwork in physical education classes motivate me to participate actively. | 5 = Strongly agree; 1 = Strongly disagree |
| Motivation for participation | PM5 | 我有动力参加体育课程，因为它们让我暂时放下学业重点，帮助我放松和恢复活力。 | I am motivated to participate in physical education classes because they offer a break from my academic focus and help me relax and rejuvenate. | 5 = Strongly agree; 1 = Strongly disagree |
| Participation consistency | PC1 | 我整个学期都坚持上体育课。 | I consistently attend my physical education classes throughout the semester. | 5 = Strongly agree; 1 = Strongly disagree |
| Participation consistency | PC2 | 我有意识地努力定期参加体育活动。 | I make a conscious effort to participate regularly in physical education activities. | 5 = Strongly agree; 1 = Strongly disagree |
| Participation consistency | PC3 | 即使在文科或理科方面的学习日程繁忙，我在体育课程中也保持稳定的参与水平。 | Even with a busy academic schedule in liberal arts or science, I maintain a steady level of participation in physical education classes. | 5 = Strongly agree; 1 = Strongly disagree |
| Participation consistency | PC4 | 我坚持自己的承诺，持续参加体育课程。 | I follow through with my commitment to participate consistently in physical education classes. | 5 = Strongly agree; 1 = Strongly disagree |
| Participation consistency | PC5 | 我定期参加体育课程，确保自己在整个课程中保持一致的参与。 | I regularly engage in scheduled physical education sessions, ensuring consistent participation throughout the course. | 5 = Strongly agree; 1 = Strongly disagree |
| Teaching equipment satisfaction | SE1 | 我对我在体育课上使用的教学设备质量感到满意。 | I am satisfied with the quality of the teaching equipment used in my physical education classes. | 5 = Highly satisfied; 1 = Highly dissatisfied |
| Teaching equipment satisfaction | SE2 | 为体育课程提供的教学设备满足了我的需要。 | The teaching equipment provided for physical education classes meets my needs. | 5 = Highly satisfied; 1 = Highly dissatisfied |
| Teaching equipment satisfaction | SE3 | 我满意于在体育课中使用的教学设备的种类和可用性。 | I am content with the variety and availability of the teaching equipment used during physical education sessions. | 5 = Highly satisfied; 1 = Highly dissatisfied |
| Teaching equipment satisfaction | SE4 | 体育课上的教学设备总体维护良好、功能齐全。 | The condition of the teaching equipment in physical education classes is generally well maintained and functional. | 5 = Highly satisfied; 1 = Highly dissatisfied |
| Teaching equipment satisfaction | SE5 | 我觉得体育教育中使用的教学设备提高了我的学习体验。 | I feel that the teaching equipment used in physical education enhances my learning experience. | 5 = Highly satisfied; 1 = Highly dissatisfied |
| Teaching venue satisfaction | SV1 | 我对海南师范大学体育教学场地的整体质量感到满意。 | I am satisfied with the overall quality of the physical education teaching venue at Hainan Normal University. | 5 = Highly satisfied; 1 = Highly dissatisfied |
| Teaching venue satisfaction | SV2 | 体育教学场地舒适，适合课堂活动。 | The physical education teaching venue is comfortable and suitable for class activities. | 5 = Highly satisfied; 1 = Highly dissatisfied |
| Teaching venue satisfaction | SV3 | 我对体育教育设施的清洁和维护感到满意。 | I am pleased with the cleanliness and maintenance of the physical education facilities. | 5 = Highly satisfied; 1 = Highly dissatisfied |
| Teaching venue satisfaction | SV4 | 体育教学场地的位置和无障碍条件符合我的需要。 | The location and accessibility of the physical education teaching venue meet my needs. | 5 = Highly satisfied; 1 = Highly dissatisfied |
| Teaching venue satisfaction | SV5 | 我觉得体育场地配备了必要的设施和资源，以支持有效的学习。 | I feel that the physical education venue is well equipped with necessary amenities and resources to support effective learning. | 5 = Highly satisfied; 1 = Highly dissatisfied |
| Teaching-method satisfaction | SM1 | 我对海南师范大学体育课上使用的教学方法感到满意。 | I am satisfied with the teaching methods used in my physical education classes at Hainan Normal University. | 5 = Highly satisfied; 1 = Highly dissatisfied |
| Teaching-method satisfaction | SM2 | 在体育课程中采用的教学方法能够有效吸引我。 | The teaching methods employed in physical education classes effectively engage me. | 5 = Highly satisfied; 1 = Highly dissatisfied |
| Teaching-method satisfaction | SM3 | 我发现体育课上的教学方法清晰易懂，增强了我的学习体验。 | I find the teaching methods in physical education classes clear and easy to understand, enhancing my learning experience. | 5 = Highly satisfied; 1 = Highly dissatisfied |
| Teaching-method satisfaction | SM4 | 体育课程中使用的各种教学方法有助于我在整个课程中保持兴趣和参与。 | The variety of teaching methods used in physical education classes helps maintain my interest and participation throughout the course. | 5 = Highly satisfied; 1 = Highly dissatisfied |
| Teaching-method satisfaction | SM5 | 我相信体育教育的教学方法非常适合适应学生不同的学习风格。 | I believe that the teaching methods in physical education are well suited to accommodate students’ diverse learning styles. | 5 = Highly satisfied; 1 = Highly dissatisfied |
| Evaluation-system satisfaction | SS1 | 我对海南师范大学体育课程中所使用的评价体系的公平性感到满意。 | I am satisfied with the fairness of the evaluation system used in my physical education classes at Hainan Normal University. | 5 = Highly satisfied; 1 = Highly dissatisfied |
| Evaluation-system satisfaction | SS2 | 用来评估我在体育教育方面表现的标准是清晰和可理解的。 | The criteria used to evaluate my performance in physical education are clear and understandable. | 5 = Highly satisfied; 1 = Highly dissatisfied |
| Evaluation-system satisfaction | SS3 | 我很满意体育教育的评价体系如何反映我的努力和进步。 | I am pleased with how the evaluation system in physical education reflects my effort and progress. | 5 = Highly satisfied; 1 = Highly dissatisfied |
| Evaluation-system satisfaction | SS4 | 体育评价系统提供的反馈帮助我提高了自己的表现和参与度。 | The feedback provided through the evaluation system in physical education helps me improve my performance and participation. | 5 = Highly satisfied; 1 = Highly dissatisfied |
| Evaluation-system satisfaction | SS5 | 我认为体育教育的评价体系是适当的，并与课程目标相关。 | I feel that the evaluation system in physical education is appropriate and relevant to the course objectives. | 5 = Highly satisfied; 1 = Highly dissatisfied |
| Physical-limitations barriers | BP1 | 我经常觉得身体上无法满足体育活动的要求。 | I often feel physically unable to keep up with the demands of physical education activities. | 5 = Strongly agree; 1 = Strongly disagree |
| Physical-limitations barriers | BP2 | 我目前的健康状况限制了我参加体育活动。 | My current health condition limits my participation in physical education. | 5 = Strongly agree; 1 = Strongly disagree |
| Physical-limitations barriers | BP3 | 由于害怕受伤，我避免参加某些体育活动。 | I avoid certain physical education activities because of fear of injury. | 5 = Strongly agree; 1 = Strongly disagree |
| Physical-limitations barriers | BP4 | 我的身体状况使我很难完全参与体育课。 | I have a physical condition that makes it difficult for me to participate fully in physical education classes. | 5 = Strongly agree; 1 = Strongly disagree |
| Physical-limitations barriers | BP5 | 我发现体育锻炼所需要的体力消耗对我的健康水平来说太有挑战性了。 | I find the physical exertion required in physical education too challenging for my fitness level. | 5 = Strongly agree; 1 = Strongly disagree |
| Time-constraint barriers | BT1 | 我的学习日程安排使我很难完全参加体育课。 | My academic schedule makes it difficult for me to participate fully in physical education classes. | 5 = Strongly agree; 1 = Strongly disagree |
| Time-constraint barriers | BT2 | 我经常在体育课和其他学术承诺之间发生冲突。 | I often have conflicts between physical education classes and other academic commitments. | 5 = Strongly agree; 1 = Strongly disagree |
| Time-constraint barriers | BT3 | 我发现平衡体育、学习时间和课外活动之间的安排具有挑战性。 | I find it challenging to balance physical education with study time and extracurricular activities. | 5 = Strongly agree; 1 = Strongly disagree |
| Time-constraint barriers | BT4 | 我在校外的个人日程安排使得我几乎没有时间积极参与体育活动。 | My personal schedule outside school leaves little time for active participation in physical education. | 5 = Strongly agree; 1 = Strongly disagree |
| Time-constraint barriers | BT5 | 体育课的时间对我的其他职责很不方便。 | The timing of physical education classes is inconvenient for my other responsibilities. | 5 = Strongly agree; 1 = Strongly disagree |
| Lack-of-interest barriers | BI1 | 我觉得体育课很无趣，因此并不想积极参与。 | I find physical education classes uninteresting and therefore do not participate actively. | 5 = Strongly agree; 1 = Strongly disagree |
| Lack-of-interest barriers | BI2 | 体育课上的运动和活动对我没有吸引力。 | The sports and activities in physical education do not appeal to me. | 5 = Strongly agree; 1 = Strongly disagree |
| Lack-of-interest barriers | BI3 | 我不认为体育对我的学术或个人目标有价值。 | I do not see the value of physical education for my academic or personal goals. | 5 = Strongly agree; 1 = Strongly disagree |
| Lack-of-interest barriers | BI4 | 我觉得自己没有动力参加体育活动，因为我对体育活动不感兴趣。 | I feel unmotivated to participate in physical education because I am not interested in physical activities. | 5 = Strongly agree; 1 = Strongly disagree |
| Lack-of-interest barriers | BI5 | 我参加体育课，但没有完全投入，因为我对体育或健身没有热情。 | I attend physical education classes but do not fully engage because I am not passionate about sports or fitness. | 5 = Strongly agree; 1 = Strongly disagree |
| Peer-related barriers | BS1 | 当别人在观看时，我对参加体育活动感到不舒服。 | I feel uncomfortable participating in physical education activities when others are watching. | 5 = Strongly agree; 1 = Strongly disagree |
| Peer-related barriers | BS2 | 我避免参加体育活动，因为我在同龄人面前感到害羞。 | I avoid participating in physical education because I feel self-conscious around my peers. | 5 = Strongly agree; 1 = Strongly disagree |
| Peer-related barriers | BS3 | 体育运动中的竞争气氛阻碍了我的积极参与。 | The competitive atmosphere in physical education discourages my active involvement. | 5 = Strongly agree; 1 = Strongly disagree |
| Peer-related barriers | BS4 | 我很少参加体育活动，因为我觉得自己没有得到同龄人的支持。 | I participate less in physical education because I do not feel supported by my peers. | 5 = Strongly agree; 1 = Strongly disagree |
| Peer-related barriers | BS5 | 体育活动中的社交互动让我感到不舒服，也限制了我的参与。 | Social interactions during physical education activities make me uncomfortable and limit my participation. | 5 = Strongly agree; 1 = Strongly disagree |

Note. English translations are provided for reporting transparency. The Chinese form is the fielded instrument. This supplementary table should be read together with the Methods section; it does not claim a formal independent forward–backward translation or cross-cultural validation.

# Appendix S1. Fielded Chinese Questionnaire Screenshots

The screenshots below document the Chinese online questionnaire used in the study. For analysis and interpretation, the scoring key in Supplementary Table S1 governs. The screenshots are archival facsimiles and should not be used to infer response coding solely from visual left-to-right label order.

|  | **Figure S2. Screenshot 2.**   |
| --- | --- |

| **Figure S3. Screenshot 3.**   | **Figure S4. Screenshot 4.**   |
| --- | --- |

| **Figure S5. Screenshot 5.**   | **Figure S6. Screenshot 6.**   |
| --- | --- |

| **Figure S7. Screenshot 7.**   |  |
| --- | --- |
