# Supplementary material for: Participation, perceived barriers, and course satisfaction in university physical education: a cross-sectional study of non-sport-major undergraduates at a Chinese university
Source: Front Psychol. 2026 Sep 9;17:1931977. doi: 10.3389/fpsyg.2026.1931977 (PMC13598082; doi:10.3389/fpsyg.2026.1931977)
Supplement: Supplementary file 2 [file Table_2.DOCX]

STROBE Checklist for Cross-Sectional Studies

**Participation and Perceived Barriers Are Associated With Physical Education Course Satisfaction Among Chinese Non-Sport-Major Undergraduates**

| **Section** | **Item** | **Recommendation** | **Location in manuscript** |
| --- | --- | --- | --- |
| Title and abstract | 1a | Indicate study design in title or abstract | Abstract; title page |
| Title and abstract | 1b | Provide an informative, balanced summary | Abstract |
| Introduction | 2 | Explain scientific background and rationale | Introduction, paragraphs 1–6 |
| Introduction | 3 | State specific objectives and hypotheses | Introduction, final paragraph |
| Methods | 4 | Present key elements of study design early | Materials and Methods: Study design and reporting |
| Methods | 5 | Describe setting, locations, and relevant dates | Materials and Methods: Setting, participants, and procedures |
| Methods | 6a | Give eligibility criteria and selection methods | Materials and Methods: Setting, participants, and procedures |
| Methods | 7 | Clearly define outcomes, exposures, predictors, and covariates | Materials and Methods: Measures |
| Methods | 8 | Describe data sources and assessment methods | Materials and Methods: Questionnaire development and Chinese administration; Measures |
| Methods | 9 | Describe efforts to address potential sources of bias | Materials and Methods: Statistical analysis; Discussion: Strengths and limitations |
| Methods | 10 | Explain how study size was arrived at | Materials and Methods: Setting, participants, and procedures [fieldwork sample retained; no a priori power calculation documented] |
| Methods | 11 | Explain how quantitative variables were handled | Materials and Methods: Measures; Statistical analysis |
| Methods | 12 | Describe statistical methods, confounding control, and sensitivity analyses | Materials and Methods: Statistical analysis; Supplementary Table S2 |
| Results | 13 | Report numbers of individuals at each stage | Results: Sample retention and data completeness; Supplementary Table S2 |
| Results | 14 | Give characteristics of study participants | Not available in final de-identified analytical archive; explicitly disclosed in Methods and Limitations |
| Results | 15 | Report outcome data | Results: Descriptive statistics and internal consistency; Tables 1–3 |
| Results | 16 | Give unadjusted and adjusted estimates and their precision | Results: Bivariate associations and regression models; Tables 2–3 |
| Results | 17 | Report other analyses | Supplementary Table S2 (data completeness and VIF diagnostics) |
| Discussion | 18 | Summarize key results with reference to objectives | Discussion: Principal findings |
| Discussion | 19 | Discuss limitations | Discussion: Strengths and limitations |
| Discussion | 20 | Provide cautious overall interpretation | Discussion; Conclusion |
| Discussion | 21 | Discuss generalizability | Discussion: Strengths and limitations |
| Other information | 22 | Give funding source and role | Funding [to be completed/confirmed] |
