# Supplementary material for: Participation, perceived barriers, and course satisfaction in university physical education: a cross-sectional study of non-sport-major undergraduates at a Chinese university
Source: Front Psychol. 2026 Sep 9;17:1931977. doi: 10.3389/fpsyg.2026.1931977 (PMC13598082; doi:10.3389/fpsyg.2026.1931977)
Supplement: Supplementary file 3 [file Table_3.DOCX]

**Revised Supplementary Statistical Tables (S3-S12)**

Supplementary Table S3. Response-option display-order and scoring audit.

| Block | Observed left-to-right display order | Intended semantic analytic coding |
| --- | --- | --- |
| Participation intensity | Strongly agree -> Agree -> Neutral -> Disagree -> Strongly disagree | 5 -> 4 -> 3 -> 2 -> 1 |
| Participation motivation | Strongly disagree -> Disagree -> Neutral -> Agree -> Strongly agree | 1 -> 2 -> 3 -> 4 -> 5 |
| Participation consistency | Strongly disagree -> Disagree -> Neutral -> Agree -> Strongly agree | 1 -> 2 -> 3 -> 4 -> 5 |
| Satisfaction: equipment | Highly dissatisfied -> Dissatisfied -> Neutral -> Satisfied -> Highly satisfied | 1 -> 2 -> 3 -> 4 -> 5 |
| Satisfaction: venue | Highly satisfied -> Satisfied -> Neutral -> Dissatisfied -> Highly dissatisfied | 5 -> 4 -> 3 -> 2 -> 1 |
| Satisfaction: teaching methods | Highly satisfied -> Satisfied -> Neutral -> Dissatisfied -> Highly dissatisfied | 5 -> 4 -> 3 -> 2 -> 1 |
| Satisfaction: evaluation | Highly dissatisfied -> Dissatisfied -> Neutral -> Satisfied -> Highly satisfied | 1 -> 2 -> 3 -> 4 -> 5 |
| Barriers: physical limitations | Strongly agree -> Agree -> Neutral -> Disagree -> Strongly disagree | 5 -> 4 -> 3 -> 2 -> 1 |
| Barriers: time constraints | Strongly disagree -> Disagree -> Neutral -> Agree -> Strongly agree | 1 -> 2 -> 3 -> 4 -> 5 |
| Barriers: lack of interest | Strongly agree -> Agree -> Neutral -> Disagree -> Strongly disagree | 5 -> 4 -> 3 -> 2 -> 1 |
| Barriers: peer-related | Strongly disagree -> Disagree -> Neutral -> Agree -> Strongly agree | 1 -> 2 -> 3 -> 4 -> 5 |

Note. Coding is defined by response meaning, not screen position. The original survey-platform button-to-code map is not preserved, so the audit cannot independently reconstruct the historical platform mapping.

Supplementary Table S4. Permutation-based parallel analysis.

| Item set/component | Observed eigenvalue | 95th-percentile permuted eigenvalue | Retain? |
| --- | --- | --- | --- |
| Participation, 1 | 6.807 | 1.485 | Yes |
| Participation, 2 | 2.533 | 1.370 | Yes |
| Participation, 3 | 1.807 | 1.292 | Yes |
| Participation, 4 | 0.875 | 1.225 | No |
| Participation, 5 | 0.579 | 1.167 | No |
| Satisfaction, 1 | 10.441 | 1.573 | Yes |
| Satisfaction, 2 | 3.046 | 1.461 | Yes |
| Satisfaction, 3 | 2.328 | 1.385 | Yes |
| Satisfaction, 4 | 1.395 | 1.316 | Yes |
| Satisfaction, 5 | 0.411 | 1.263 | No |
| Satisfaction, 6 | 0.319 | 1.212 | No |
| Perceived barriers, 1 | 8.667 | 1.572 | Yes |
| Perceived barriers, 2 | 2.091 | 1.458 | Yes |
| Perceived barriers, 3 | 1.713 | 1.380 | Yes |
| Perceived barriers, 4 | 1.614 | 1.317 | Yes |
| Perceived barriers, 5 | 0.700 | 1.261 | No |
| Perceived barriers, 6 | 0.602 | 1.208 | No |

Note. Parallel analysis used 1,000 independent column permutations. The 95th-percentile criterion retained 3 participation factors, 4 satisfaction factors, and 4 perceived-barrier factors.

Supplementary Table S5. Rotated pattern matrix for PE participation.

| Item | Intended domain | F1 | F2 | F3 |
| --- | --- | --- | --- | --- |
| 1. I regularly attend my Physical Education classes as part of my program. | Intensity | 0.663 | -0.097 | 0.036 |
| 2. During Physical Education classes, I actively participate in activities and exercises, regardless of whether they align with my major. | Intensity | 0.854 | -0.025 | -0.040 |
| 3. I put in consistent effort during Physical Education sessions, even though my primary focus is on other subjects. | Intensity | 0.917 | -0.024 | 0.020 |
| 4. I engage fully in Physical Education activities, understanding their importance despite my major. | Intensity | 0.799 | 0.058 | 0.014 |
| 5. I am committed to improving my physical fitness through active participation in Physical Education. | Intensity | 0.701 | 0.088 | -0.013 |
| 6. I am motivated to participate in Physical Education classes because I believe it contributes to my overall well-being. | Motivation | -0.072 | 0.888 | 0.021 |
| 7. The skills and knowledge I gain in Physical Education classes motivate me to actively participate. | Motivation | 0.021 | 0.912 | -0.011 |
| 8. I participate in Physical Education classes because I enjoy the physical activities. | Motivation | 0.093 | 0.848 | 0.016 |
| 9. The social interactions and teamwork in Physical Education classes motivate me to engage actively. | Motivation | 0.010 | 0.898 | 0.004 |
| 10. I am motivated to participate in Physical Education classes because they offer a break from my academic focus and help me relax and rejuvenate. | Motivation | -0.042 | 0.923 | -0.013 |
| 11. I consistently attend my Physical Education classes throughout the semester. | Consistency | -0.060 | 0.085 | 0.579 |
| 12. I make a conscious effort to participate regularly in Physical Education activities. | Consistency | 0.249 | 0.159 | 0.520 |
| 13. Even with a busy academic schedule, I maintain a steady level of participation in Physical Education classes. | Consistency | 0.189 | 0.167 | 0.536 |
| 14. I follow through with my commitment to participate in Physical Education classes consistently. | Consistency | -0.029 | -0.058 | 0.952 |
| 15. I regularly engage in Physical Education sessions as scheduled, ensuring that my participation is consistent throughout the course. | Consistency | -0.019 | 0.003 | 0.898 |

Factor correlations: F1-F2 = 0.363; F1-F3 = 0.379; F2-F3 = 0.451.

Note. Principal-axis factoring with oblimin rotation. Pattern coefficients are shown; factor signs and order were aligned with the intended domains for interpretability.

Supplementary Table S6. Rotated pattern matrix for PE course satisfaction.

| Item | Intended domain | F1 | F2 | F3 | F4 |
| --- | --- | --- | --- | --- | --- |
| 1. I am satisfied with the quality of the teaching equipment used in my Physical Education classes. | Equipment | 0.878 | -0.009 | 0.037 | -0.029 |
| 2. The teaching equipment provided for Physical Education classes meets my needs. | Equipment | 0.856 | 0.040 | 0.008 | 0.024 |
| 3. I am content with the variety and availability of the teaching equipment used during Physical Education sessions. | Equipment | 0.958 | -0.030 | 0.035 | -0.026 |
| 4. The condition of the teaching equipment in Physical Education classes is generally well-maintained and functional. | Equipment | 0.914 | 0.061 | -0.067 | -0.013 |
| 5. I feel that the teaching equipment used in Physical Education enhances my learning experience. | Equipment | 0.828 | -0.031 | 0.009 | 0.092 |
| 6. I am satisfied with the overall quality of the Physical Education teaching venue at Hainan Normal University. | Venue | -0.019 | 0.905 | -0.020 | 0.008 |
| 7. The Physical Education teaching venue is comfortable and suitable for the activities conducted in class. | Venue | -0.030 | 0.924 | 0.000 | 0.000 |
| 8. I am pleased with the cleanliness and maintenance of the Physical Education facilities. | Venue | 0.102 | 0.794 | -0.047 | -0.021 |
| 9. The location and accessibility of the Physical Education teaching venue meet my needs. | Venue | 0.022 | 0.852 | 0.057 | 0.004 |
| 10. I feel that the Physical Education venue is well-equipped with necessary amenities and resources to support effective learning. | Venue | -0.015 | 0.838 | 0.043 | 0.030 |
| 11. I am satisfied with the teaching methods used in my Physical Education classes at Hainan Normal University. | Teaching methods | -0.026 | 0.052 | 0.886 | -0.013 |
| 12. The teaching methods employed in Physical Education classes effectively engage me. | Teaching methods | 0.019 | 0.048 | 0.912 | -0.055 |
| 13. I find the teaching methods in Physical Education classes clear and easy to understand, enhancing my learning experience. | Teaching methods | -0.005 | -0.036 | 0.970 | 0.008 |
| 14. The variety of teaching methods used in Physical Education classes helps maintain my interest and participation throughout the course. | Teaching methods | 0.014 | -0.003 | 0.913 | 0.010 |
| 15. I believe that the teaching methods in Physical Education are well-suited to accommodate diverse learning styles. | Teaching methods | 0.015 | -0.023 | 0.883 | 0.069 |
| 16. I am satisfied with the fairness of the evaluation system used in my Physical Education classes at Hainan Normal University. | Evaluation | 0.034 | -0.003 | 0.021 | 0.874 |
| 17. The criteria used for evaluating my performance in Physical Education are clear and understandable. | Evaluation | 0.036 | -0.010 | 0.005 | 0.920 |
| 18. I am pleased with how the evaluation system in Physical Education reflects my efforts and progress. | Evaluation | 0.001 | -0.022 | 0.025 | 0.952 |
| 19. The feedback provided through the evaluation system in Physical Education helps me improve my performance and participation. | Evaluation | -0.025 | 0.028 | 0.004 | 0.942 |
| 20. I feel that the evaluation system in Physical Education is appropriate and relevant to the objectives of the course. | Evaluation | -0.023 | 0.021 | -0.037 | 0.966 |

Factor correlations: F1-F2 = 0.629; F1-F3 = 0.450; F1-F4 = 0.416; F2-F3 = 0.578; F2-F4 = 0.350; F3-F4 = 0.478.

Note. Principal-axis factoring with oblimin rotation. Pattern coefficients are shown; factor signs and order were aligned with the intended domains for interpretability.

Supplementary Table S7. Rotated pattern matrix for perceived barriers.

| Item | Intended domain | F1 | F2 | F3 | F4 |
| --- | --- | --- | --- | --- | --- |
| 1. I often feel physically unable to keep up with the demands of PE activities. | Physical limitations | 0.780 | -0.055 | 0.019 | 0.075 |
| 2. My current health condition limits my participation in PE. | Physical limitations | 0.858 | 0.042 | -0.010 | -0.034 |
| 3. I avoid certain PE activities due to fear of injury. | Physical limitations | 0.631 | -0.056 | -0.012 | 0.068 |
| 4. I have a physical condition that makes it difficult for me to fully participate in PE classes. | Physical limitations | 0.627 | 0.124 | 0.091 | -0.001 |
| 5. I find the physical exertion required in PE too challenging for my fitness level. | Physical limitations | 0.688 | 0.056 | 0.052 | 0.072 |
| 6. My academic schedule makes it difficult for me to fully participate in PE classes. | Time constraints | -0.001 | 0.799 | 0.017 | 0.044 |
| 7. I often have conflicts between PE classes and other academic commitments. | Time constraints | -0.010 | 0.837 | 0.020 | -0.017 |
| 8. I find it challenging to balance PE with my study time and extracurricular activities. | Time constraints | 0.120 | 0.692 | -0.079 | -0.045 |
| 9. My personal schedule outside of school leaves little time for active participation in PE. | Time constraints | 0.068 | 0.730 | -0.009 | -0.030 |
| 10. The timing of PE classes is inconvenient for my other responsibilities. | Time constraints | -0.069 | 0.781 | 0.079 | 0.084 |
| 11. I find PE classes uninteresting and therefore do not participate actively. | Lack of interest | -0.065 | -0.025 | 0.844 | 0.045 |
| 12. The sports and activities in PE do not appeal to me. | Lack of interest | -0.084 | 0.024 | 0.873 | 0.033 |
| 13. I do not see the value of PE for my academic or personal goals. | Lack of interest | -0.009 | 0.083 | 0.681 | 0.034 |
| 14. I feel unmotivated to participate in PE because I am not interested in physical activities. | Lack of interest | 0.163 | 0.011 | 0.821 | -0.072 |
| 15. I attend PE classes but do not fully engage because I am not passionate about sports or fitness. | Lack of interest | 0.133 | 0.020 | 0.735 | 0.022 |
| 16. I feel uncomfortable participating in PE activities when others are watching. | Peer-related | 0.041 | -0.110 | -0.024 | 0.742 |
| 17. I avoid participating in PE because I feel self-conscious around my peers. | Peer-related | 0.039 | 0.056 | -0.020 | 0.756 |
| 18. The competitive atmosphere in PE discourages me from active involvement. | Peer-related | -0.004 | 0.090 | -0.094 | 0.810 |
| 19. I participate less in PE because I do not feel supported by my peers. | Peer-related | 0.028 | 0.027 | 0.143 | 0.691 |
| 20. Social interactions during PE activities make me uncomfortable and limit my participation. | Peer-related | 0.011 | -0.019 | 0.088 | 0.787 |

Factor correlations: F1-F2 = 0.496; F1-F3 = 0.510; F1-F4 = 0.495; F2-F3 = 0.535; F2-F4 = 0.420; F3-F4 = 0.476.

Note. Principal-axis factoring with oblimin rotation. Pattern coefficients are shown; factor signs and order were aligned with the intended domains for interpretability.

Supplementary Table S8. Sensitivity analyses for Model 2.

| Predictor | OLS-HC3 B [95% CI] | 3,000-bootstrap 95% CI | Huber B | Median-regression B |
| --- | --- | --- | --- | --- |
| Participation | 0.472 [0.351, 0.592]; p < .001 | [0.353, 0.589] | 0.509; p < .001 | 0.580; p < .001 |
| Physical limitations | 0.077 [-0.039, 0.194]; p = .194 | [-0.035, 0.192] | 0.048; p = .240 | 0.057; p = .231 |
| Time constraints | -0.180 [-0.288, -0.073]; p = .001 | [-0.289, -0.081] | -0.170; p < .001 | -0.182; p < .001 |
| Lack of interest | -0.182 [-0.310, -0.055]; p = .005 | [-0.298, -0.046] | -0.157; p = .002 | -0.146; p = .013 |
| Peer-related barriers | -0.009 [-0.105, 0.088]; p = .861 | [-0.102, 0.084] | -0.025; p = .528 | -0.007; p = .877 |

Supplementary Table S9. Domain-level participation sensitivity model.

| Predictor | B | 95% CI | p |
| --- | --- | --- | --- |
| Participation intensity | 0.255 | [0.116, 0.394] | < .001 |
| Participation motivation | 0.100 | [0.015, 0.186] | .021 |
| Participation consistency | 0.166 | [0.050, 0.283] | .005 |
| Physical limitations | 0.077 | [-0.037, 0.191] | .186 |
| Time constraints | -0.170 | [-0.277, -0.062] | .002 |
| Lack of interest | -0.186 | [-0.308, -0.064] | .003 |
| Peer-related barriers | -0.004 | [-0.101, 0.093] | .938 |

Note. Outcome = broad course-satisfaction composite. HC3 robust standard errors. R² = .451; adjusted R² = .437.

Supplementary Table S10. Spearman correlations among intended domain scores.

| Domain set | Pair | Spearman’s ρ |
| --- | --- | --- |
| Participation | Intensity – Motivation | 0.583 |
| Participation | Intensity – Consistency | 0.584 |
| Participation | Motivation – Consistency | 0.590 |
| Satisfaction | Equipment – Venue | 0.677 |
| Satisfaction | Equipment – Methods | 0.553 |
| Satisfaction | Equipment – Evaluation | 0.566 |
| Satisfaction | Venue – Methods | 0.570 |
| Satisfaction | Venue – Evaluation | 0.495 |
| Satisfaction | Methods – Evaluation | 0.658 |
| Perceived barriers | Physical – Time | 0.487 |
| Perceived barriers | Physical – Interest | 0.534 |
| Perceived barriers | Physical – Peer | 0.516 |
| Perceived barriers | Time – Interest | 0.539 |
| Perceived barriers | Time – Peer | 0.440 |
| Perceived barriers | Interest – Peer | 0.540 |

Note. All domain correlations are positive and statistically significant at p < .001. These correlations describe relatedness among the intended domains; they do not establish unidimensionality of the broad composites.

Supplementary Table S11. Sensitivity models using each satisfaction domain as the outcome.

| Outcome | Predictor | B | 95% CI | p |
| --- | --- | --- | --- | --- |
| Equipment | Participation | 0.588 | [0.384, 0.791] | < .001 |
| Equipment | Physical limitations | 0.117 | [-0.042, 0.275] | .148 |
| Equipment | Time constraints | -0.141 | [-0.307, 0.025] | .095 |
| Equipment | Lack of interest | -0.173 | [-0.376, 0.031] | .096 |
| Equipment | Peer-related barriers | -0.054 | [-0.211, 0.103] | .499 |
| Venue | Participation | 0.352 | [0.165, 0.539] | < .001 |
| Venue | Physical limitations | 0.120 | [-0.047, 0.287] | .158 |
| Venue | Time constraints | -0.266 | [-0.436, -0.095] | .002 |
| Venue | Lack of interest | -0.197 | [-0.427, 0.033] | .093 |
| Venue | Peer-related barriers | -0.006 | [-0.182, 0.171] | .949 |
| Methods | Participation | 0.475 | [0.283, 0.667] | < .001 |
| Methods | Physical limitations | 0.095 | [-0.055, 0.245] | .214 |
| Methods | Time constraints | -0.215 | [-0.352, -0.078] | .002 |
| Methods | Lack of interest | -0.218 | [-0.457, 0.021] | .074 |
| Methods | Peer-related barriers | 0.038 | [-0.097, 0.173] | .584 |
| Evaluation | Participation | 0.472 | [0.258, 0.685] | < .001 |
| Evaluation | Physical limitations | -0.023 | [-0.201, 0.155] | .801 |
| Evaluation | Time constraints | -0.100 | [-0.221, 0.022] | .108 |
| Evaluation | Lack of interest | -0.142 | [-0.345, 0.061] | .171 |
| Evaluation | Peer-related barriers | -0.012 | [-0.154, 0.130] | .865 |

Note. Each row comes from an HC3-robust OLS model containing broad PE participation plus the four barrier domains. Participation was positively associated with all four satisfaction domains. Time constraints were negatively associated with venue and teaching-method satisfaction. Estimates for lack of interest were negative for all four domains, but their individual 95% CIs included zero. These results show that the broad satisfaction association pattern is not identical across all course-satisfaction facets.

Supplementary Table S12. Verified record-level exclusion-log summary.

| Primary exclusion reason | n | % of excluded (N = 50) | % of distributed (N = 350) |
| --- | --- | --- | --- |
| Incomplete questionnaire / missing key questions | 12 | 24.0 | 3.4 |
| Abnormally short completion time | 10 | 20.0 | 2.9 |
| Pronounced straight-lining / extremely low response variability | 8 | 16.0 | 2.3 |
| Duplicate submission | 6 | 12.0 | 1.7 |
| Failure to meet eligibility criteria | 6 | 12.0 | 1.7 |
| Clearly mechanical / patterned responding | 4 | 8.0 | 1.1 |
| Irreconcilable screening inconsistencies / invalid records | 2 | 4.0 | 0.6 |
| Other incomplete or unusable records | 2 | 4.0 | 0.6 |
| Total excluded | 50 | 100.0 | 14.3 |

Note. During revision, the research team recovered and verified a record-level exclusion log covering all 50 nonretained questionnaires. The table reports one primary exclusion reason per record. The excluded questionnaires and original screening paradata are not contained in the de-identified analytical archive; therefore, the current reanalysis did not retrospectively re-estimate or modify the historical screening thresholds.
